# Aminoacyl-tRNA synthetases in human health and disease

**DOI:** 10.3389/fphys.2022.1029218

**Published:** 2022-10-18

**Authors:** Alexandra K. Turvey, Gabriella A. Horvath, André R. O. Cavalcanti

**Affiliations:** ^1^ Department of Biology, Pomona College, Claremont, CA, United States; ^2^ Division of Biochemical Genetics, Department of Pediatrics, University of British Columbia, BC Children’s Hospital, Vancouver, BC, Canada; ^3^ Adult Metabolic Diseases Clinic, Vancouver General Hospital, Vancouver, BC, Canada

**Keywords:** aminoacyl-tRNA synthetases (aaRSs), genetic diseases, human health, charcot-marie-tooth (CMT) disease, rare disease

## Abstract

The Aminoacyl-tRNA Synthetases (aaRSs) are an evolutionarily ancient family of enzymes that catalyze the esterification reaction linking a transfer RNA (tRNA) with its cognate amino acid matching the anticodon triplet of the tRNA. Proper functioning of the aaRSs to create aminoacylated (or “charged”) tRNAs is required for efficient and accurate protein synthesis. Beyond their basic canonical function in protein biosynthesis, aaRSs have a surprisingly diverse array of non-canonical functions that are actively being defined. The human genome contains 37 genes that encode unique aaRS proteins. To date, 56 human genetic diseases caused by damaging variants in aaRS genes have been described: 46 are autosomal recessive biallelic disorders and 10 are autosomal dominant monoallelic disorders. Our appreciation of human diseases caused by damaging genetic variants in the aaRSs has been greatly accelerated by the advent of next-generation sequencing, with 89% of these gene discoveries made since 2010. In addition to these genetic disorders of the aaRSs, anti-synthetase syndrome (ASSD) is a rare autoimmune inflammatory myopathy that involves the production of autoantibodies that disrupt aaRS proteins. This review provides an overview of the basic biology of aaRS proteins and describes the rapidly growing list of human diseases known to be caused by genetic variants or autoimmune targeting that affect both the canonical and non-canonical functions of these essential proteins.

## 1 Introduction

The Central Dogma of molecular biology explains the flow of genetic information in a biological system from DNA through RNA to proteins (Crick, 1970). The Aminoacyl-tRNA Synthetases (aaRSs)—a family of enzymes present in all eukaryotes, archaea, and bacteria—link the worlds of nucleic acids and proteins and are key for the faithful translation of the genetic code (Kaiser et al., 2020). aaRSs catalyze the esterification that links a transfer RNA (tRNA) with its cognate amino acid matching the anticodon triplet of the tRNA (Ibba and Söll, 2000; Rubio Gomez and Ibba, 2020). aaRSs are evolutionarily ancient, emerging during the time of the last universal common ancestor (LUCA), and are distributed across all branches of life.

**FIGURE 1:**
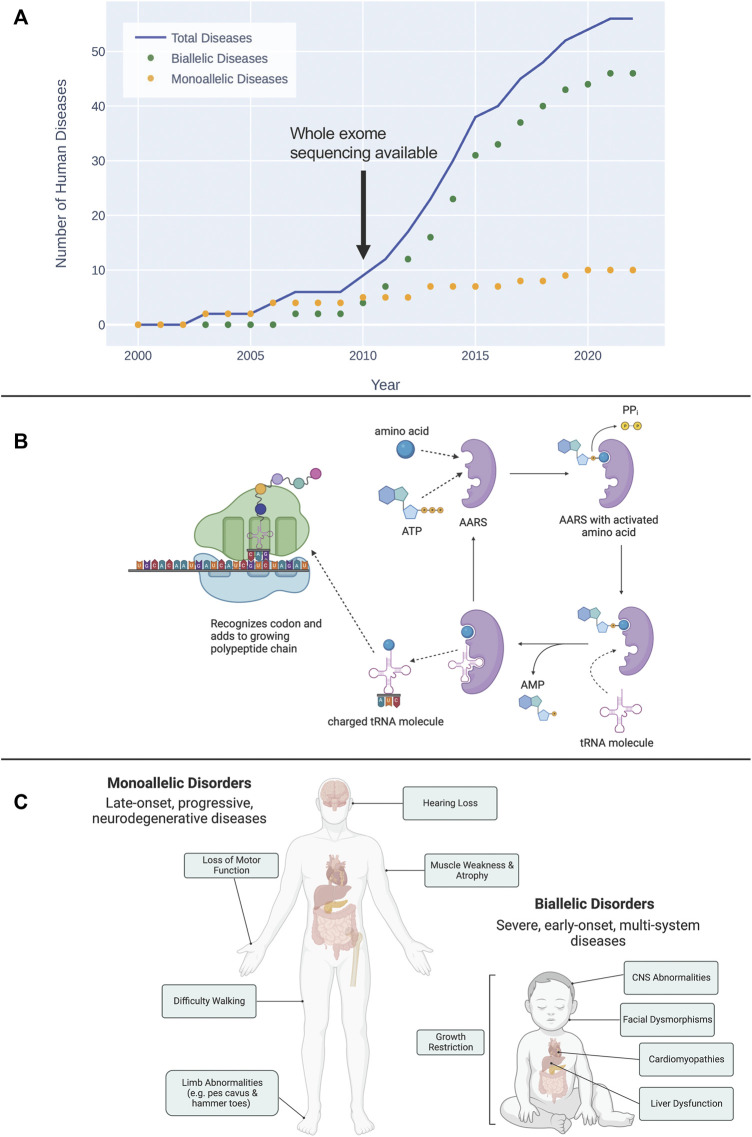
**(A)** The timeline of discovery of biallelic and monoallelic human diseases caused by damaging aaRS variants since 2000. **(B)** Overview of the esterification reaction, catalyzed by aaRS proteins, that charges a tRNA molecule with its cognate amino acid. This charged tRNA then travels to a translating ribosome where it recognizes a codon and adds its amino acid to the growing polypeptide chain. **(C)** General characterization of symptoms associated with diseases caused by monoallelic and biallelic pathogenic aaRS variants. Diseases caused by monoallelic variants are typically late-onset, progressive, neurodegenerative disorders. Damaging biallelic variants cause severe, early-onset, multi-system disorders.

Given their central role in human biology, it is unsurprising that genetic variants that disrupt aaRS protein structure and function cause disease. Damaging variants in aaRSs have now been linked to over 50 human diseases (Figure 1A). Notably 2/3 of these newly recognized diseases have only been described in the past decade since next-generation sequencing technologies have become more widely available. This review will provide an overview of the basic biology of aaRS proteins and will describe the rapidly growing list of human diseases known to be caused by variants that affect both the canonical and non-canonical functions of these essential proteins. We anticipate this review will be of value to clinicians who care for patients with diseases related to aaRS function, and scientists interested in the links between the aaRSs and disease.

## 2 Overview of basic biological functions of the aaRSs

### 2.1 Canonical function

The synthesis of functional proteins relies on the accurate transfer of genetic information from DNA, through mRNA, to protein. To generate new proteins, DNA is first transcribed to mRNA using the complementarity of nucleotide bases. The mRNA is then translated into a protein by the ribosome. The ribosome reads the mRNA in groups of triplet codons with each codon corresponding to one amino acid.

Once the ribosome reads a codon, elongation factors bring a tRNA containing the three bases complementary to a codon (anticodon) and charged with the correct amino acid. The amino acid delivered by the tRNA is then added to the growing polypeptide chain following the order of codons specified by the mRNA. This elongating polypeptide eventually becomes a functional protein within the cell.

The sequence of amino acids in a protein is defined by what amino acid is brought to the ribosome by the tRNA cognate to each codon. The essential step in this process involves linking the appropriate amino acid with its matching tRNA. This is the reaction catalyzed by the aaRSs, the esterification that links each tRNA molecule with its cognate amino acid creating an aminoacylated, or “charged” tRNA (Figure 1B). In addition to the aminoacylation functions, during evolution some tRNA synthetases added an editing function to remove the wrong amino acid from its cognate tRNA (Ling et al., 2009).

With few exceptions, all living organisms contain genes for the 20 aaRSs, one for each amino acid. The degeneracy of the genetic code means that there are more codons than amino acids. Therefore, the aaRS for an amino acid can recognize several tRNAs cognate to that amino acid. Eukaryotes contain genes for cytoplasmic and mitochondrial aaRSs (and in plants, chloroplastic aaRSs). All of these proteins are nuclear encoded and synthesized in the cytoplasm with the organellar aaRSs being imported to their final destinations following translation.

In humans and other higher eukaryotes, 9 of the aaRS proteins (ArgRS, AspRS, GlnRS, GluRS, IleRS, LeuRS, LysRS, MetRS, and ProRS) bind together to form the cytoplasmic multi-tRNA synthetase complex (MSC) (Kerjan et al., 1994; Havrylenko and Mirande, 2015; Khan et al., 2022). While the exact cellular function of the MSC is still unknown (Cui et al., 2021), it has been proposed that the MSC may act to enhance translation efficiency by channeling charged tRNAs to the A-site of a protein-synthesizing ribosome (Kyriacou and Deutscher, 2008; Khan et al., 2020; Sissler, 2021).

### 2.2 Non-canonical functions

During evolution, most eukaryotic cytoplasmic aaRSs gained noncatalytic domains not found in their respective orthologs in bacteria or archaea (Guo et al., 2010a; 2010b; Yao and Fox, 2020). These additions correlate with the progressive complexity of eukaryotes. In most cases, these additional domains are dispensable for aminoacylation or editing, suggesting a role beyond the ‘housekeeping’ function of aaRSs in protein translation. aaRSs have been recognized to have a surprisingly diverse array of non-canonical (or ‘moonlighting’) functions involved in gene expression regulation, RNA splicing, tumorigenesis, angiogenesis, and the immune responses (Ivanov et al., 2000; Smirnova et al., 2012). While it is beyond the scope of this review to address all non-canonical functions, the well-characterized ‘moonlighting’ human tyrosyl tRNA synthetase (TyrRS) serves as an illustrative example. TyrRS has no cell signaling capacity as a full molecule but when split by proteolysis each fragment can act as a cytokine (Wakasugi and Schimmel, 1999). For example, mini-TyrRS, the N-terminal domain of TyrRS, is released by endothelial cells and exhibits angiogenic and leukocyte chemoattractant properties (Wakasugi et al., 2002).

Our understanding of these non-canonical functions of aaRSs is growing rapidly. As we try to understand how pathogenic genetic changes in the aaRS genes cause human disease, it is essential that we look beyond the canonical aminoacylation role of aaRSs and consider how alterations in non-canonical functions may also contribute to pathophysiology (Guo et al., 2010b).

## 3 aaRS nomenclature and the exceptions

aaRS nomenclature is somewhat complex as it integrates functional classes, subcellular localization, and gene names. Here we clarify and summarize this nomenclature. First, aaRS proteins are divided into two classes based on their specific structural and functional properties (Cusack et al., 1990; Eriani et al., 1990). Class I aaRSs contain two highly conserved sequence motifs (‘KMSKS’ and ‘HIGH’) that are part of the larger conserved Rossman fold domain (Eriani et al., 1990; Shepard et al., 1992). Class II aaRSs, on the other hand, are less conserved than the Class I enzymes and contain a unique alpha-beta fold in their catalytic domains (Bullwinkle and Ibba, 2014; Smith and Hartman, 2015). Although the two aaRS classes are evolutionarily and structurally very different, the overall chemistry of the tRNA aminoacylation reaction is similar in both—an example of convergent evolution (Arnez and Moras, 1997).

The naming convention for genes encoding aaRSs is as follows: 1) gene names begin with the one-letter symbol for the amino acid the aaRS recognizes (e.g., *A* for alanine; *Y* for tyrosine); 2) this is followed by *ARS1* or *ARS2* depending on whether the aaRS is located in the cytoplasm or mitochondria, respectively. For example, *LARS2* specifies leucyl-tRNA synthetase 2, which charges a tRNA molecule with leucine in the mitochondria of the cell.

While the single-letter amino acid code is used for aaRS gene names, the 3-letter amino acid code is generally used as a prefix to refer to the protein product. For example, IleRS refers to isoleucyl-tRNA synthetase.

The majority of genes encoding aaRSs follow the naming convention described above, but there are a few exceptions. *EPRS1* encodes a bifunctional glutamyl-prolyl-tRNA synthetase that has the capacity to charge tRNA molecules with either glutamic acid or proline in the cytoplasm (Cerini et al., 1991; Jin et al., 2021). The mitochondrial glutaminyl-tRNA synthetase is not encoded for by a separate gene in mammalian species, and it has instead been proposed that an indirect pathway allows for the synthesis of GlnRS in mammalian mitochondria (Nagao et al., 2009). Both *KARS1* and *GARS1* encode for synthetases that dually-localize to both the mitochondria and cytoplasm (Yao and Fox, 2013). Finally, the cytoplasmic phenylalanine-tRNA synthetase consists of a heterodimer of two protein subunits: *FARSA* encodes the catalytic alpha subunit while *FARSB* encodes the regulatory beta subunit (Rodova et al., 1999).

In total, human cells contain 37 genes that encode unique aaRS proteins. Of these, 18 encode cytoplasmic aaRSs (2 of these genes encode cytoplasmic PheRS), 17 encode mitochondrial synthetases, and two genes encode proteins that will exist in both locations (Wei et al., 2019).

## 4 Human diseases associated with genetic variants in aaRS-encoding genes

Next-generation sequencing (NGS) technology has transformed our ability to make genetic diagnoses. Since the first successful application of NGS for gene identification in 2010 (Ng et al., 2010), the discovery of human diseases caused by pathogenic genetic variants has rapidly increased (Bamshad et al., 2019). It is anticipated that this number will continue to grow with improvements in both sequencing technologies and bioinformatic tools to pinpoint pathogenic variants (Schuler et al., 2022). Throughout this review we will use the term ‘variant’ to describe a change in the germline DNA sequence, as it has been recommended to replace the terms ‘mutation’ and ‘polymorphism’ with the term ‘variant’ (Richards et al., 2015).

To date, 56 human diseases caused by damaging variants in aaRS genes have been described (see Figure 1A). Emphasizing the diagnostic impact of NGS, 89% of these gene discoveries were made since 2010. Of these 56 diseases, 46 are autosomal recessive and are caused by damaging biallelic variants, while the remaining 10 are autosomal dominant and are caused by damaging monoallelic variants (Figure 1C). Biallelic disease occurs when there is a pathogenic variant on both alleles of a given gene, whereas a monoallelic disease is caused by a pathogenic variant affecting one of the two alleles. Biallelic disease can follow two possible inheritance patterns: 1) the same damaging variant occurs on both alleles (homozygous inheritance); and 2) unique damaging variants occur on each allele (compound heterozygous inheritance). The 56 human diseases linked to genetic variation in the aaRSs have been shown to span all three of these possible inheritance patterns (see Table 1).

**TABLE 1:** Complete list of each aaRS-encoding gene and every human disease in the Online Mendelian Inheritance in Man resource (OMIM - https://www.omim.org) caused by damaging monoallelic and biallelic variants in that gene.

aaRS gene name	Protein name	Monoallelic diseases		Biallelic diseases	
		Disease name (OMIM number)	References	Disease name (OMIM number)	References
*AARS1*	Alanyl-tRNA synthetase 1	Leukoencephalopathy, hereditary diffuse, with spheroids 2 (#619661)	Sundal et al. (2019)	Developmental and epileptic encephalopathy 29 (#616339)	Simons et al. (2015)
		Charcot-Marie-Tooth disease, axonal, type 2N (#613287)	Latour et al. (2010)	Trichothiodystrophy 8, nonphotosensitive (#619691)	Botta et al. (2021)
*AARS2*	Alanyl-tRNA synthetase 2			Combined oxidative phosphorylation deficiency 8 (#614096)	Götz et al. (2011)
				Leukoencephalopathy, progressive, with ovarian failure (#615889)	Dallabona et al. (2014)
*CARS1*	Cysteinyl-tRNA synthetase 1			Microcephaly, developmental delay, and brittle hair syndrome (#618891)	Kuo et al. (2019)
*CARS2*	Cysteinyl-tRNA synthetase 2			Combined oxidative phosphorylation deficiency 27 (#616672)	Hallmann et al. (2014)
*DARS1*	Aspartyl-tRNA synthetase 1			Hypomyelination with brainstem and spinal cord involvement and leg spasticity (#615281)	Taft et al. (2013)
*DARS2*	Aspartyl-tRNA synthetase 2			Leukoencephalopathy with brain stem and spinal cord involvement and lactate elevation (#611105)	Scheper et al. (2007)
*EARS2*	Glutamyl- tRNA synthetase 2			Combined oxidative phosphorylation deficiency 12 (#614924)	Steenweg et al. (2012)
*EPRS1*	Glutamyl-prolyl-tRNA synthetase 1			Leukodystrophy, hypomyelinating, 15 (#617951)	Mendes et al. (2018)
*FARSA*	Phenylalanyl-tRNA synthetase a			Rajab interstitial lung disease with brain calcifications 2 (#619013)	Krenke et al. (2019)
*FARSB*	Phenylalanyl-tRNA synthetase b			Rajab interstitial lung disease with brain calcifications 1 (#613658)	Antonellis et al. (2018)
*FARS2*	Phenylalanyl-tRNA synthetase 2			Combined oxidative phosphorylation deficiency 14 (#614946)	Shamseldin et al. (2012)
*GARS1*	Glycyl-tRNA synthetase 1 (both cytoplasmic and mitochondrial)	Charcot-Marie-Tooth disease, type 2D (#601472)	Antonellis et al. (2003)		
		Neuronopathy, distal hereditary motor, type VA (#600794)	Antonellis et al. (2003)		
		Spinal muscular atrophy, infantile, James type (#619042)	James et al. (2006)		
*HARS1*	Histidyl-tRNA synthetase 1	Charcot-Marie-Tooth disease, axonal, type 2W (#616625)	Vester et al. (2013)	Usher syndrome type 3 B (#614504)	Puffenberger et al. (2012)
*HARS2*	Histidyl-tRNA synthetase 2			Perrault syndrome 2 (#614926)	Pierce et al. (2011)
*IARS1*	Isoleucine-tRNA synthetase 1			Growth retardation, impaired intellectual development, hypotonia, and hepatopathy (#617093)	Kopajtich et al. (2016)
*IARS2*	Isoleucine-tRNA synthetase 2			Cataracts, growth hormone deficiency, sensory neuropathy, sensorineural hearing loss, and skeletal dysplasia (#616007)	Schwartzentruber et al. (2014)
*KARS1*	Lysyl-tRNA synthetase (both cytoplasmic and mitochondrial)			Charcot-Marie-Tooth disease, recessive intermediate, B (#613641)	McLaughlin et al. (2010)
				Deafness, autosomal recessive 89 (#613916)	Santos-Cortez et al. (2013)
				Deafness, congenital, and adult-onset progressive leukoencephalopathy (#619196)	Zhou et al. (2017)
				Leukoencephalopathy, progressive, infantile-onset, with or without deafness (#619147)	McMillan et al. (2015)
*LARS1*	Leucyl-tRNA synthetase 1			Infantile liver failure syndrome 1 (#615438)	Casey et al. (2012)
*LARS2*	Leucyl-tRNA synthetase 2			Perrault syndrome 4 (#615300)	Pierce et al. (2013)
				Hydrops, lactic acidosis, and sideroblastic anemia (#617021)	Riley et al. (2016)
*MARS1*	Methionyl-tRNA synthetase 1	Charcot-Marie-Tooth disease, axonal, type 2U (#616280)	Gonzalez et al. (2013)	Trichothiodystrophy 9, nonphotosensitive (#619692)	Botta et al. (2021)
				Interstitial lung and liver disease (#615486)	van Meel et al. (2013)
*MARS2*	Methionyl-tRNA synthetase 2			Combined oxidative phosphorylation deficiency 25 (#616430)	Webb et al. (2015)
				Spastic ataxia 3, autosomal recessive (#611390)	Bayat et al. (2012)
*NARS1*	Asparaginyl-tRNA synthetase 1	Neurodevelopmental disorder with microcephaly, impaired language, epilepsy, and gait abnormalities, autosomal dominant (#619092)	Manole et al. (2020), Wang et al. (2020)	Neurodevelopmental disorder with microcephaly, impaired language, and gait abnormalities, autosomal recessive (#619091)	Manole et al. (2020)
*NARS2*	Asparaginyl-tRNA synthetase 2			Deafness, autosomal recessive 94 (#618434)	Simon et al. (2015)
				Combined oxidative phosphorylation deficiency 24 (#616239)	Vanlander et al. (2015)
*PARS2*	Prolyl-tRNA synthetase 2			Developmental and epileptic encephalopathy 75 (#618437)	Sofou et al. (2015)
*QARS1*	Glutaminyl- tRNA synthetase 1			Microcephaly, progressive, seizures, and cerebral and cerebellar atrophy (#615760)	Zhang et al. (2014)
*RARS1*	Arginyl-tRNA synthetase 1			Leukodystrophy, hypomyelinating, 9 (#616140)	Wolf et al. (2014)
*RARS2*	Arginyl-tRNA synthetase 2			Pontocerebellar hypoplasia, type 6 (#611523)	Edvardson et al. (2007)
*SARS1*	Seryl-tRNA synthetase 1			Neurodevelopmental disorder with microcephaly, ataxia, and seizures (#617709)	Musante et al. (2017)
*SARS2*	Seryl-tRNA synthetase 2			Hyperuricemia, pulmonary hypertension, renal failure, and alkalosis (#613845)	Belostotsky et al. (2011)
*TARS1*	Threonyl-tRNA synthetase 1			Trichothiodystrophy 7, nonphotosensitive (#618546)	Theil et al. (2019)
*TARS2*	Threonyl-tRNA synthetase 2			Combined oxidative phosphorylation deficiency 21 (#615918)	Diodato et al. (2014)
*VARS1*	Valyl-tRNA synthetase 1			Neurodevelopmental disorder with microcephaly, seizures, and cortical atrophy (#617802)	Karaca et al. (2015)
*VARS2*	Valyl-tRNA synthetase 2			Combined oxidative phosphorylation deficiency 20 (#615917)	Taylor et al. (2014)
*WARS1*	Tryptophanyl-tRNA synthetase 1	Neuronopathy, distal hereditary motor, type IX (#617721)	Tsai et al. (2017)		
*WARS2*	Tryptophanyl-tRNA synthetase 2			Neurodevelopmental disorder, mitochondrial, with abnormal movements and lactic acidosis, with or without seizures (#617710)	Musante et al. (2017)
				Parkinsonism-dystonia 3, childhood-onset (#619738)	Burke et al. (2018)
*YARS1*	Tyrosyl-tRNA synthetase 1	Charcot-Marie-Tooth disease, dominant intermediate C (#608323)	Jordanova et al. (2006)	Infantile-onset multisystem neurologic, endocrine, and pancreatic disease 2 (#619418)	Nowaczyk et al. (2017)
*YARS2*	Tyrosyl-tRNA synthetase 2			Myopathy, lactic acidosis, and sideroblastic anemia 2 (#613561)	Riley et al. (2010)

### 4.1 Monoallelic diseases

To date, 10 monoallelic diseases arising from autosomal dominant variants in seven aaRS genes (*NARS1*, *HARS1*, *GARS1*, *AARS1*, *MARS1*, *WARS1*, *YARS1*) have been identified (see Table 1). Interestingly, all of these monoallelic conditions occur in cytoplasmic AARS-encoding genes.

#### 4.1.1 Charcot-marie-tooth disease

The most common monoallelic condition associated with AARS variants is Charcot-Marie-Tooth (CMT) disease. CMT is a clinically and genetically heterogeneous neurodegenerative disorder that affects the peripheral nervous system in roughly 1 in 2,500 individuals (Skre, 1974; Blocquel et al., 2019). Symptoms typically arise in early adulthood, manifesting as the progressive loss of motor and sensory functions. Clinical features include progressive weakness and atrophy in distal muscles leading to motor impairment, areflexia, limb abnormalities (esp. foot deformities), and a range of sensory loss (Skre, 1974; Rossor et al., 2013; Bansagi et al., 2015; Wei et al., 2019). There are currently no curative options for CMT. Treatment is supportive and focuses on maximizing function through physical and occupational therapies, orthopedic devices such as braces, and sometimes orthopedic surgery. Pain relief medications are used for CMT patients who experience severe pain. However, as the genetics of CMT are defined, therapies addressing the underlying molecular dysfunction are being developed (Pisciotta et al., 2021).

CMT is divided into various subtypes, with demyelinating Type 1 CMT (CMT1) and axonal Type 2 CMT (CMT2) containing the majority of cases. Demyelinating Type 1 CMT occurs from breakdown of the myelin sheath of nerves, while Type 2 axonal CMT occurs from direct damage to the axons of nerves (Harding and Thomas, 1980; Bird, 1993; Teunissen et al., 2003). Intermediate CMT has features of both Type 1 and Type 2.

While over 90 genes have been linked to the pathogenesis of CMT, aaRS-encoding genes constitute the largest gene family connected with this disorder (Bansagi et al., 2015; Blocquel et al., 2019). So far, variants in seven cytoplasmic aaRS genes have been established to cause CMT (*YARS1*, *MARS1*, *KARS1, WARS1*, *AARS1*, *GARS1*, *HARS1*). All of these genes except *YARS1* and *KARS1* are associated with monoallelic axonal CMT (CMT2). *YARS1* causes dominant intermediate C CMT. *KARS1* is an outlier in that it causes autosomal recessive CMT, and specifically the intermediate B subtype.

CMT is specifically associated with monoallelic pathogenic variants in cytoplasmic aaRSs, indicating a special sensitivity of the peripheral nervous system to this type of genetic change. Given the estimate that only ∼20% of the CMT-related variants affect canonical catalytic function (Datt and Sharma, 2014), the simple loss of aminoacylation activity is not a prerequisite for disease (Storkebaum et al., 2009; Froelich and First, 2011; Niehues et al., 2016; Zhang et al., 2021). Much work has been done to try to understand how monoallelic variants in cytoplasmic aaRSs cause disease in an autosomal dominant fashion and here we will highlight some key themes. Because the aaRS proteins are essential for protein synthesis in every cell, the challenge has been to determine if the pathogenic variants result in disease because they impair general protein synthesis or whether the disease causing aaRS variants become toxic to normal cellular function. The general experimental strategy has been to over express the wild-type version of the aaRS in an animal model of CMT disease (e.g., WT GlyRS in the dominant mouse models of CMT2D (Motley et al., 2011)). This experimental strategy can then test the hypothesis that if the disease is caused by a loss of function, then the overexpression would rescue the disease phenotype. In general, these types of genetic engineering studies in various model organisms (i.e., mice, flies, worms, and fish) have confirmed the dominant toxicity of pathogenic aaRS variants known to cause CMT (reviewed in (Wei et al., 2019)).

In recent studies, a complementary pair of papers by Zuko *et al.* and Spaulding *et al.* expanded our understanding of disease mechanisms in CMT (Mellado and Willis, 2021; Spaulding et al., 2021; Zuko et al., 2021). The investigators found that a disease causing GlyRS variant bound tRNA^Gly^ but failed to release it. This sequestration likely reduced the cellular tRNA^Gly^ pool, leading to insufficient tRNA^Gly^ supply to the ribosome. Indeed, they observed ribosome stalling at glycine codons and chronic activation of the damaging integrated stress response (ISR) in affected motor neurons through the sensor kinase GCN2. Importantly, these complementary studies identified two strategies with potential therapeutic benefit: 1) overexpression of tRNA^Gly^ to rescue protein synthesis, avoiding ISR activation and the ensuing peripheral neuropathy; or 2) inhibiting GCN2 to avoid activation of the neurotoxic ISR. Currently, the mechanism(s) linking aaRS variants to human CMT remains an area of intense investigation with the ultimate goal of developing treatments that will prevent neurodegeneration in patients born with aaRS variants that cause CMT.

#### 4.1.2 Other monoallelic neurological disorders

While CMT is the most common monoallelic condition associated with aaRS variants, several other related neurological conditions are caused by damaging aaRS variants. It is helpful to appreciate that borders between these disease definitions are rather ‘porous’ and that these additional monoallelic aaRS-related conditions share features with CMT and with each other.

Distal hereditary motor neuronopathy (dHMN) is a pure motor neuropathy characterized by progressive distal muscle weakness and muscular atrophy without sensory impairment. Pathogenic variants in both *WARS1* and *GARS1* have been associated with dHMN (Antonellis et al., 2003; Tsai et al., 2017).

Neurodevelopmental disorder with microcephaly, impaired language, epilepsy, and gait abnormalities (designated NEDMILEG) is reported to be caused by *de novo* heterozygous variants in the *NARS1* gene (Manole et al., 2020). Notably, bi-allelic variants in *NARS1* also cause a similar neurodevelopmental disease. The mechanism of disease for the *de novo* heterozygous variants was suggested to be toxic gain-of-function, while the bi-allelic recessive variants were thought to cause disease through partial loss-of-function.

James type of infantile spinal muscular atrophy (SMAJI) is a severe neuromuscular disorder with symptoms beginning in the first weeks or months of life. Several unrelated children with SMAJI have been found to have *de novo* heterozygous variants in *GARS1* (James et al., 2006; Eskuri et al., 2012; Forrester et al., 2020; Markovitz et al., 2020).

### 4.2 Biallelic diseases

Biallelic diseases arising from the disruption of both alleles of genes encoding the aaRSs cause severe, early-onset disorders affecting multiple organ systems. Biallelic disease can be caused by homozygous or compound heterozygous variants.

Autosomal recessive aaRS deficiencies represent a rapidly growing group of severe inherited diseases (Figure 1A) involving multiple organ systems and currently without curative treatment options. Fuchs et al. (2019) recently analyzed symptoms across aaRS biallelic disorders and found that the most common features of these disorders are: central nervous system (CNS) abnormalities, growth restriction, liver dysfunction, and facial dysmorphisms (Fuchs et al., 2019).

Current treatment options for autosomal recessive aaRS deficiencies are very limited. However, functional studies on variants that cause biallelic disease have demonstrated a reduction of the relevant aaRS protein level and/or decreases in aminoacylation enzymatic activity (Meyer-Schuman and Antonellis, 2017; Kok et al., 2021). Importantly, these patients still have some intrinsic aminoacylation activity. It is hypothesized that deficiencies in the aaRS enzymes may result in the inability to supply sufficient charged tRNAs to support protein synthesis, especially during periods of increased demand, such as rapid growth and infections (Kok et al., 2022). Knowledge of this disease mechanism led Kok *et al.* to trial a personalized intervention in four patients based on oral supplementation with the cognate amino acid matching the patients’ aaRS deficiency (e.g., the patient with biallelic *IARS1* variants received high doses of oral l-isoleucine) (Kok et al., 2021). This amino acid supplementation was well-tolerated and safe, and showed encouraging results in terms of improvements in growth, development, and ability to cope with intercurrent infections. It is anticipated that this result will encourage more trials in additional patients to more formally assess the safety and efficacy of this treatment approach which targets the underlying aminoacylation defect in patients with autosomal recessive aaRS deficiencies.

Biallelic aaRS diseases are multi-system disorders with significant cross-over between phenotypes. It is likely that more clarity will emerge around the clinical phenotypes as more patients and more genetic diagnoses are described. Indeed, Fuchs *et al.* emphasized the importance of deep phenotyping of patients with aaRS-related diseases and reporting all clinical features, so the full extent of the phenotypes can be appreciated (Fuchs et al., 2019). For simplicity we have divided this section into biallelic disorders affecting mitochondrial or cytoplasmic aaRSs.

#### 4.2.1 Biallelic disorders affecting mitochondrial aaRSs

All aaRS proteins are nuclear-encoded enzymes. After their translation in the cytosol, the mitochondrial aaRSs must be imported into the mitochondrial matrix to perform their canonical role of charging mitochondrial genome-encoded tRNA molecules with their cognate amino acids. Essential cellular processes rely on available ATP, the cellular energy currency, which is generated by oxidative phosphorylation that takes place in the five respiratory complexes in the mitochondria (Sissler et al., 2017; González-Serrano et al., 2019). AaRS proteins play a role in mitochondrial oxidative phosphorylation because accurate translation of the 13 mitochondrial-encoded proteins involved in oxidative phosphorylation and ATP production requires properly functioning mitochondrial aaRS proteins (Fine et al., 2019).

Combined oxidative phosphorylation deficiency (COXPD) is a unifying umbrella term describing a large group of multisystem disorders caused by defects in the mitochondrial oxidative phosphorylation system. Currently more than 50 different types of COXPD have been described, each caused by damaging variants in genes critical to the integrity of mitochondrial oxidative phosphorylation. The most common biallelic disease category associated with mitochondrial aaRSs is COXPD. To date, eight mitochondrial aaRS-encoding genes have been found to cause various forms of COXPD (*MARS2*, *CARS2*, *EARS2*, *VARS2*, *TARS2, AARS2*, *FARS2*, *NARS2*). All forms of this COXPD caused by pathogenic aaRS variants cause damage to the CNS (which requires an abundant and constant energy supply) (Moulinier et al., 2017; Sissler et al., 2017; Zheng et al., 2022). Other common features include liver disease, visual impairment, and microcephaly. COXPD8, which arises from variants in the *AARS2* gene, has been shown to cause lethal cardiomyopathy (Götz et al., 2011; Taylor et al., 2014).

Beyond the umbrella term of COXPD, when viewed in aggregate, biallelic disorders of the mitochondrial aaRSs predominantly cause disease of the central nervous system (i.e. leukoencephalopathies, epilepsy, developmental delay, intellectual disability, sensorial neural hearing loss). However, other organs systems are also affected, manifesting as liver disease, myopathies, and ovarian failure (Meyer-Schuman and Antonellis, 2017). Hence, not all biallelic disorders of the mitochondrial aaRSs cause disease that falls cleanly under the broad COXPD phenotype. For example, damaging biallelic variants in *HARS2* and *LARS2* cause Perrault syndrome, an autosomal recessive disorder characterized by sensorineural deafness in both males and females, and ovarian dysgenesis in affected females (Pierce et al., 2011; Pierce et al., 2013). While there remains much to learn about these disorders, the fact that biallelic defects in mitochondrial aaRS enzymes do not all lead to identical phenotypes suggests that the underlying disease mechanisms might involve alterations in non-canonical ‘moonlighting’ function rather than solely defects in aminoacylation (Roux et al., 2021).

#### 4.2.2 Biallelic disorders affecting cytosolic aaRSs

Biallelic variants in the cytosolic aaRSs predominantly cause neurological disease, notably leukodystrophies, leukoencephalopathies, and other neurodevelopmental disorders. Leukodystrophies are genetic disorders affecting the white matter of the CNS with or without peripheral nervous system involvement. Genetic leukoencephalopathies refer to related neurological conditions with significant white matter abnormalities that do not meet criteria for inclusion as a leukodystrophy (Vanderver et al., 2015). Leukodystrophies and leukoencephalopathies both profoundly impact the CNS, causing abnormalities and degeneration of cerebral white matter (Kaye and Moser, 2004; Parikh et al., 2015; Tang et al., 2019). Biallelic cytosolic aaRS variants are known to cause both leukoencephalopathies (linked to *KARS1*) and leukodystrophies (*RARS1*, *EPRS1*). Interestingly, a damaging monoallelic variant in the *AARS1* gene has also been found to cause leukoencephalopathy in two members of an affected Swedish family (Sundal et al., 2019). Other neurodevelopmental disorders have been linked to three biallelic variants (*VARS1*, *SARS1*, *NARS1*) and one monoallelic variant (*NARS1*) in cytosolic aaRS genes. Symptoms of these disorders include intellectual disability, delayed language development and ability to walk, microcephaly, movement disorders, and in some cases seizures (Hübers et al., 2020). There is clear cross-over between the manifestations of the disorders caused by biallelic mitochondrial and cytosolic aaRS variants which will only be resolved through the sequencing and careful phenotyping of more affected individuals.

## 5 Anti-synthetase syndrome

Autoimmune diseases are the result of the body inappropriately mounting an immune response against itself. In addition to the genetic disorders of the aaRSs, there is also an acquired autoimmune condition affecting aaRSs, called Anti-Synthetase Syndrome (ASSD). ASSD is a rare condition that involves the production of autoantibodies that bind with, and mount a response against, aaRS proteins (Kron and Härtlein, 2013; Galindo-Feria et al., 2022). ASSD is an idiopathic inflammatory myopathy with organ complications beyond the muscles, including interstitial lung disease (Mahler et al., 2014). ASSD has features that overlap with dermatomyositis and polymyositis (Lepreux et al., 2018). The formal diagnostic criteria for ASSD are based on the presence of anti-aminoacyl tRNA synthetase antibodies along with major (interstitial lung disease and/or polymyositis or dermatomyositis) and minor (arthritis, Raynaud’s phenomenon, mechanic’s hands) criteria (Solomon et al., 2011; Witt et al., 2016). The most commonly recognized autoantigen is HisRS (recognized by anti-Jo-1 autoantibodies), but to date, autoantibodies targeting eight aaRSs (HisRS, ThrRS, AlaRS, GlyRS, IleRS, AsnRS, PheRS, TyrRS) have been linked to ASSD (Ascherman, 2015; Galindo-Feria et al., 2022). A combination of immunosuppressive agents is used to treat ASSD (Witt et al., 2016). While our understanding of the pathophysiology of ASSD remains incomplete, autoimmune targeting of the aaRSs may trigger their non-canonical immune functions to activate the innate and adaptive immunity (Gallay et al., 2018).

## 6 Discussion and future directions

The recent rapid advances in defining the role of aaRSs in human disease opens many avenues for life changing improvements in diagnosis and targeted treatment. The ability to provide a complete genetic diagnosis for individuals with monoallelic or biallelic aaRS diseases is transformative in many ways—new treatments can be explored based on the genetic findings (i.e., personalized medicine), new potential medical issues or risks can be anticipated and avoided, and accurate genetic counselling can be provided for the patient and their extended family. Ultimately, our current understanding of aaRSs in health and disease represents a powerful integration of knowledge that has emerged through the study of evolutionary science, basic biochemistry, and clinical medicine.
